# Nurse Navigator of Cancer Patients: contributions to the discussion on the national stage

**DOI:** 10.1590/0034-7167.2024770201

**Published:** 2024-02-26

**Authors:** Edvane Birelo Lopes de Domenico, Luciana Puchalski Kalinke

**Affiliations:** I Universidade Federal de São Paulo. São Paulo, São Paulo, Brazil; II Universidade Federal do Paraná. Curitiba, Paraná, Brazil

The professional practice of patient navigation is incipient in Brazil and, therefore, a broad discussion with various sectors of society is essential, which is the main purpose of this editorial. Shall we start with the story.

Patient navigation was defined in 1989 as a community-based service delivery intervention aimed at appropriately promoting access to diagnosis and treatment of cancer and other chronic diseases by eliminating hindering barriers. The concept was defined based on hearings promoted by the American Cancer Society (USA) in seven North American cities, in which mainly people with cancer in conditions of social vulnerability participated^(1)^.

The first cancer patient navigation program was designed and implemented by doctor Harold Freeman, in 1990, in Harlem, New York, USA. Freeman & Rodriguez described barriers to be eliminated or minimized in the navigation program, namely: financial and access, such as lack of or problems with health insurance; health system; communication and information; and emotional barriers, such as fear and distrust. As for determining who should navigate, the authors attributed this decision to the intrinsic correspondence of the level of skills required at each phase of navigation of the care continuum^(1)^.

Starting in 2007, the Oncology Nursing Society (ONS, USA) sought to pave the way for cancer patient navigation as an area of practice for oncology nurses, highlighting the importance of these nurses, working in navigation programs, assessing the impact on the concept under development. Both the ONS and other oncology nurse societies in high-income countries have invested in defining competencies and designing structured models for navigation programs. The search for well-designed studies, which normally came from national literature reviews, contributed to building the phenomena and subsequent validation processes with specialist professionals and scholars in the field^(2)^.

The positive impact of instituting nurse-led cancer patient navigation programs has been an inexorable reality internationally. Different assessment perspectives, including reducing waiting time between referrals-consultations-procedures, better use of therapeutic and technological resources, better clinical responses and patient satisfaction, among other gains, properly characterize personand family-centered care, even though there has been heterogeneity in the design and implementation of services over the last three decades^(3)^.

Given the evidence, we need to build the national story of navigation programs for cancer patients. Our epidemiological and social vulnerability indicators or even low health literacy produce results of unsatisfactory adherence to care to promote and maintain Brazilian health, and greatly characterize barriers to timely and quality cancer care. Furthermore, we have, geographically, an immense inequality in the supply and resources of oncological care at all levels of care. Certainly, navigation programs designed for each segment of the cancer illness process are necessary and will characterize the core competencies for each phase of navigation.

It is time to support nurses with solid oncology training to undertake navigation programs in Brazil. However, we emphasize that program designs must articulate the available scientific literature, with diagnostic studies of the reality of cancer care they experience, negotiating infrastructure and processes with managers, agreeing on assignments with the multidisciplinary team and establishing navigation activities and results goals to be achieved.

Certainly, it is the right of oncologist nurses, dedicated to patient navigation programs, to be professionally recognized as having yet another area of specialty, since the *lato sensu* graduate degree comprises the incorporation of technical skills and the ability to develop a new professional profile. It also makes it possible to improve performance in the world of work and meet demands for more technically qualified professionals, both for the public sector and for companies and third sector organizations, with a view to the country’s development^(4)^.

The training of this specialist may consist of learning guidelines for the care of patients with chronic non-communicable diseases, with multidimensional health models (biopsychosocial and spiritual), clinical and health education strategies, including the premises of health literacy, public policies for Brazilian oncology care, with an emphasis on the adoption of lines of care in both public and supplementary health as well as models and strategies for managing resources, people and performance and quality indicators. The science of nursing itself, with its theories, models and processes, cannot be removed from the training of oncologist nurses and navigators of patients and their families in the care continuum; in this scenario, it is necessary to rescue classic models and add contemporary ones.

The challenges arising from experience in the new specialty are already encouraging Brazilian specialist nurses to uncover problems or generate effective interventions through scientific research linked to *Stricto Sensu* Graduate Programs in professional or academic modalities. The scientific evidence generated by research will, over time, have the potential to refine professional skills and navigation programs, led by oncologist nurses at the national level, strengthening them.

Therefore, let us move forward with greater training resolution for oncologist nurses, with recognized practical experience, so that they acquire the knowledge, skills and attitudes necessary to take over the coordination of care and/or leadership of navigation programs, creating a new and promising area specialty.
